# Encounters and management of oral conditions at general medical practices in Australia

**DOI:** 10.1186/s12913-022-08299-2

**Published:** 2022-08-08

**Authors:** An-Lun Cheng, Joerg Eberhard, Julie Gordon, Madhan Balasubramanian, Amber Willink, Woosung Sohn, Jennifer Dai, Christopher Harrison

**Affiliations:** 1 School of Dentistry, University of Sydney, Camperdown, Australia; 2 Charles Perkins Centre, University of Sydney, Camperdown, Australia; 3 School of Health Sciences, University of Sydney, Camperdown, Australia; 4 Menzies Centre for Health Policy and Economics, University of Sydney, Camperdown, Australia; 5 Health Care Management, College of Business, Government and Law, Flinders University, Adelaide, Australia; 6 Westmead Clinical School, University of Sydney, Camperdown, Australia

**Keywords:** Oral health, Primary care, General practitioner, Health services, Health policy

## Abstract

**Background:**

Poor oral health has been widely recognised as an ongoing public health issue. Patients with oral conditions may visit either a general practitioner (GP) or a dental practitioner for management. The aims of this study are to report (i) the GP management rate of oral health conditions by patient and GP demographics, (ii) what specific oral conditions were managed, and (iii) how GPs managed oral conditions.

**Methods:**

Data from the Bettering the Evaluation and Care of Health study (2006 to 2016 inclusive) were analysed. Descriptive statistics with 95% confidence intervals around point estimates were used to summarise data. Multivariate logistic regression was performed to determine the independent effect of patient and GP characteristics.

**Results:**

A total of 972,100 GP encounters were included in the dataset, with oral condition-related encounters managed at a rate of 1.19 oral conditions per 100 GP encounters. Patients who were aged 54 years or younger, resided in a socioeconomically disadvantaged area, came from a non-English speaking background or Indigenous background were more likely to have oral conditions managed by GPs. The most commonly reported oral conditions were dental and oral mucosa-related. Over 60% of oral conditions were managed by GPs through prescribed medications.

**Conclusions:**

This study provided an overview of management of oral conditions by GPs in Australia. Patients from certain vulnerable demographic groups were more likely to attend a GP for management of oral conditions. Common oral conditions and management approaches were identified. The findings of this study contribute to public health and health policy discussions around optimising primary care provision in oral health.

**Supplementary Information:**

The online version contains supplementary material available at 10.1186/s12913-022-08299-2.

## Background

Oral health is a widely neglected global public health issue [1]. The Global Burden of Disease Study 2017 estimated 3.5 billion cases of oral conditions globally, with majority of these conditions being untreated caries and severe periodontitis [1]. These conditions are frequently accompanied by pain and impaired function, leading to interference with daily activities and influencing overall health [2]. Poor oral health has been shown to have negative effects on other health conditions including cardiovascular disease and diabetes [3, 4]. Undoubtedly, the recognition and effective management of oral conditions are imperative to alleviate this ongoing public health challenge.

Barriers that prevent people with oral conditions from attaining professional management of their conditions include limited public funded dental care, out of pocket expenses, poor accessibility, and dental fear or anxiety [5, 6]. In most countries, there is an unequal distribution of oral health services between urban and rural areas and a strong relationship between poor oral health and disadvantaged socioeconomic status (SES) [7, 8]. A lack of accessible public oral health services may also prohibit people from low socioeconomic backgrounds in acquiring proper services to manage their oral conditions [5]. These barriers to accessing oral health services may potentially redirect individuals to consider treatment from alternative medical services, such as general practitioners (GP) and hospital emergency departments (ED).

Medical services may bring improved accessibility for provision of treatment of oral conditions [9, 10]. A 2010 study among adults in the state of Maryland, US demonstrated that 14.3% visited a medical doctor for dental problems [11]. A study from Canada conducted a secondary data analysis, which revealed an average of 1.3 visits per 100 patients per year were made to physicians for oral condition-related diagnoses between 2001 and 2011 [12]. A similar secondary data analysis conducted in the United Kingdom (UK) demonstrated an average of 0.6 visits per 100 patients per year to a GP for dental consultations between 2004 and 2013 [13]. More recently, a qualitative study on Irish dentist’s perspective on integrating oral health care in primary medical care demonstrated that medical practitioners may be involved in effective collaborative oral health care if provided adequate training and education [14]. Due to the emerging number of studies worldwide on patients seeking medical services for oral conditions, there has been an increase in recognition of the potential role of a primary care medical practitioners in relieving oral health disparities [11, 15, 16].

Despite the prevalence of preventable oral disease, it has been routinely separated from primary medical care with limited collaboration between primary medical practitioners and dental practitioners [17]. More recently, oral health in the context of integrated healthcare has been an emerging topic. Primary care pathways for improving oral health in vulnerable population groups were highlighted in recent Australian studies [18–21]. Furthermore, collaborative healthcare models have been integrated in Australian community oral health programs, involving multidisciplinary health professionals such as doctors, nurses, and dieticians [22, 23]. Despite the emphasis on the importance of integrated oral healthcare by recent studies, there is a lack of literature on the types of oral conditions managed in primary care settings and how these conditions are managed. Moreover, little is known about the demographic characteristics of patients who receive oral health care in primary care settings, which may facilitate recognition of traits in patients who are more likely to present oral conditions to GPs and traits in GPs who are more likely to manage oral conditions. Such information may allow for development of public health initiatives to improve the oral health care provided by GPs to vulnerable population groups.

The aims of this study are to describe (i) the demographics of patients who had oral conditions managed by a GP from 2006 to 2016; (ii) the demographics of GPs who managed these oral conditions; (iii) the type and rate of oral conditions managed by GPs and (iv) how GPs managed these oral conditions.

## Methods

### Data collection and source

Data for the study were sourced from the last ten years (2006–16) of the Bettering the Evaluation and Care of Health (BEACH) study, a continuous cross-sectional study of general practice activity conducted from 1998–2016. The BEACH methods have been described in detail elsewhere [24]. In brief, the BEACH data captured general practice clinical activity from an ever-changing, random sample of approximately 1,000 Australian GPs each year. Each GP participant recorded details of 100 consecutive encounters with patients’ informed consent.

### Data items and management

Encounter details included patient characteristics, up to three reasons for encounter, up to four problems managed, new or follow-up problem status and method of management. Management actions were recorded as medications (inclusive of prescribed, supplied and advised for over-the-counter purchase), therapeutic procedures, clinical treatments, referrals, pathology and imaging tests ordered. Each management action was explicitly linked to the specific problem or diagnosis managed. New problems were defined as the first presentation of a problem or previously-resolved recurrent problem.

Patients’ reasons for encounter, problems or diagnoses managed and non-pharmacological management actions were coded according to ICPC-2 PLUS, an interface terminology classified to the International Classification of Primary Care—Version 2 (ICPC-2) [25]. The Anatomic Therapeutic Chemical (ATC) classification was used to classify medications among recorded management details [26]. The Index of Relative Socio-Economic Advantage and Disadvantage (IRSAD) was used to record patient relative socioeconomic status based on patient residential post code [27]. Scores 1–5 were classified as socio-economically advantaged while scores 6–10 were classified as socio-economically disadvantaged. The geographic location of the GP practice was classified using the Australian Statistical Geography Standard (ASGS) [28]. For this study, the groups Outer regional, Remote and Very remote were grouped into one group called Outer regional/Remote. Patient Indigenous status was determined by whether they self-identified as Aboriginal and/or Torres Strait Islander. Patient language background was determined by whether the patient spoke a language other than English at home.

Other patient characteristics recorded include sex, age and Health Care Concession Card (HCC) status. Reported GP characteristics include sex, age, practice size by number of GPs at practice and by full-time equivalent GPs at practice, and country of graduation. Oral problems or diagnoses by ICPC-2 PLUS code derived from GPs’ encounter reports may have overlapping or similar definitions. To provide meaningful stratification, oral problems or diagnoses (by ICPC-2 PLUS code) were grouped into non-overlapping main groups of conditions including dental, oral mucosal (oral soft tissues including mucosa, tongue and palate), periodontal (gum conditions), temporomandibular joint, oral glands, trauma, neuralgia, and dental related conditions upon discussion between all co-authors. These conditions were then classified into sub-groups to provide greater detail (Additional file 1: Appendix A).

### Data analysis

The BEACH study had a single stage cluster design with each GP (sampling unit) having a cluster of 100 patient encounters (unit of inference) around them. To adjust for this cluster, robust 95% confidence intervals (CIs) were calculated using survey procedures in SAS 9.4. Statistical significance of difference between point estimates was determined by non-overlapping 95% CIs. This method is more conservative than the usual 5% level, reducing the risk of type I errors, while increasing the risk of type II errors [29]. To determine the independent effect of patient and GP characteristics, a multivariate logistic regression was performed using the GP and patient characteristics collected using the survey logistic procedure in SAS 9.4 which also took clustering into account.

### Ethics

The BEACH program has ethics approval from the Human Research Ethics Committee of the University of Sydney (reference no.: 2012/130) and the Australian Institute of Health and Welfare (AIHW) Ethics Committee for the relevant years of collaboration (2006–11).

## Results

### Patient characteristics

A total of 9,721 GPs recorded 972,100 patient encounters, over the 10-year period of the BEACH study. During the observation period, 11,546 oral conditions were managed at a rate of 1.19 oral conditions per 100 encounters (Table 1). When extrapolated to the 143 million MBS GP items of service claimed in 2015–16[24], we estimate that there were 1,684,000 oral health conditions managed in general practice that year. People aged 70 years or older had a significantly lower oral condition management rate than in those aged 54 years and younger. Significantly higher GP management rates of oral conditions were reported in patients living in socioeconomically disadvantaged areas, HCC holders, patients from non-English speaking backgrounds, and Aboriginal and Torres Strait Islander patients (Table 1).

The multiple logistic regression model showed that patients aged 25–39 years were the most likely to have oral conditions managed at GP encounters, approximately 54% more likely compared to patients aged 70 years or above (Table 1). The modelling also revealed a higher likelihood of having oral conditions managed by GPs for patients who lived in socioeconomically disadvantaged areas (6% more likely), Concession card holders (49% more likely), patients from non-English speaking backgrounds (31% more likely), and Aboriginal and Torres Strait Islander patients (87% more likely) compared to their reference groups.

### GP characteristics

Management rates of oral conditions by GP characteristics are depicted in Table 1. GPs working in solo GP practices (1.38 per 100 encounters) or in a practice with 15 or more GPs (1.24 per 100 encounters) were significantly more likely to manage an oral health condition at an encounter than those working in practices with 2–14 GPs (1.12 to 1.19 per 100 encounters). The multiple logistic regression model identified practice location and practice size as independent predictors of the number of oral conditions managed by GPs per 100 encounters. An oral condition was 13% more likely to be managed at an encounter in a major city compared to outer regional/remote areas. A solo GP was 12–15% more likely to manage an oral condition at encounter than those working with other GPs.

### Oral condition related encounter

**Fig. 1:**
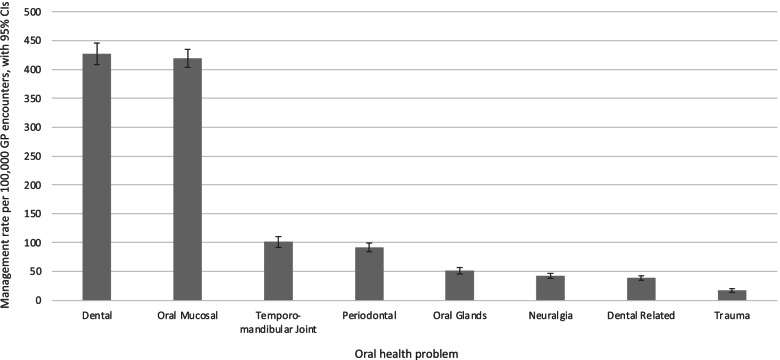
Management rate of oral conditions per 100,000 GP encounters by definition of oral problem from 2006 to 2016

Among the types of oral conditions, dental and oral mucosa (soft tissues including mucosa, tongue and palate) related problems had the highest management rates of 426.8 and 419.2 conditions per 100,000 encounters respectively. Management rates of temporomandibular joint related problems and periodontal conditions were respectively managed at a rate of 101.0 and 91.2 problems per 100,000 encounters respectively. Other oral conditions including oral gland problems, neuralgia, trauma, and other dental problems (such as teething and problems related to dental prostheses) were managed at low rates of less than 50 problems managed per 100,000 encounters (Fig. 1). Among the oral conditions specifically reported in the present study, dental infection or disease, oral ulceration, oral fungal infection, and dental pain or symptoms were commonly recorded by GPs.

### Management actions used for oral condition by GPs

Frequencies and proportions of oral conditions managed with specific management approaches are outlined in Table 1. About 62.2% of all oral conditions were managed by GPs with one or more medications, which included antibiotics (30.2%), analgesics (14.4%), stomatological preparations including topical antifungal and steroid creams (10.0%), antimycotics (3.9%), anti-inflammatory and antirheumatics (4.2%) and antiepileptics (1.7%). The next most commonly reported management action used to manage oral conditions were referrals (21.4%) (mostly to dentists (14.7%)) followed by and counselling, advice or education (14.9%). Investigations such as pathology (5.5%) and imaging (5.3%) were infrequently used while procedural/physical treatments were rarely used (2.3%).

**Table 1:** GP and patient specific management rates of oral conditions, 2006 to 2016

	N (n = 972,100)	Number of oral conditions managed	Distribution (%) of oral conditions managed (95% CI)	Characteristic specific oral conditions managed per 100 encounters (95% CI)	Odds ratios (multiple logistic regression) (95% CIs)
**Patient Characteristics**					
Sex					p = 0.1759
* Missing*	*8,522*	*114*			
Male	391,152	4,585	40.1% (39.1-41.1)	1.17 (1.14-1.21)	Reference group
Female	572,426	6,847	59.9% (58.9-60.9)	1.20 (1.17-1.23)	1.031
Age					p < 0.0001
* Missing*	*19,222*	*194*			
0-14 years	110,864	1378	12.1% (11.5-12.8)	1.24 (1.17-1.31)	1.45 (1.29-1.64)
15-24 years	81,201	1,017	9.0% (8.4-9.5)	1.25 (1.17-1.33)	1.45 (1.28-1.64)
25-39 years	148,287	1,891	16.7% (15.9-17.4)	1.28 (1.21-1.34)	1.54 (1.37-1.73)
40-54 years	186,787	2,426	21.4% (20.6-22.1)	1.30 (1.24-1.35)	1.53 (1.36-1.72)
55-69 years	206,331	2,365	20.8% (20.0-21.6)	1.15 (1.10-1.19)	1.27 (1.14-1.42)
70-84 years	170,665	1,782	15.7% (15.0-16.4)	1.04 (0.99-1.10)	1.00 (0.90-1.12)
85+ years	48,743	493	4.3% (3.9-4.8)	1.01 (0.91-1.11)	Reference Group
Socioeconomic status					p = 0.0251
* Missing*	*22,692*	*266*			
Advantaged	573,803	6,449	57.2% (55.9-58.4)	1.12 (1.09-1.15)	Reference Group
Disadvantaged	375,605	4,831	42.8% (41.6-44.1)	1.29 (1.25-1.33)	1.06 (1.01-1.11)
Concession Card Status					p < 0.0001
* Missing*	*80,058*	*823*			
Cardholder	396,992	5,518	51.5% (50.4-52.5)	1.39 (1.35-1.43)	1.49 (1.42-1.56)
Non-cardholder	495,050	5,205	48.5% (47.5-49.6)	1.05 (1.02-1.08)	Reference Group
Language background					p < 0.0001
* Missing*	*95,865*	*976*			
Non-English speaking	74,672	1,198	11.3% (10.5-12.2)	1.60 (1.50-1.71)	1.31 (1.22-1.41)
English speaking	801,563	9,372	88.7% (87.8-89.5)	1.17 (1.14-1.20)	Reference Group
Indigenous status					p < 0.0001
* Missing*	*95,622*	*978*			
Indigenous	14,791	369	3.5% (3.0-4.0)	2.49 (2.22-2.77)	1.87 (1.65-2.12)
Non-Indigenous	861,687	10,199	96.5% (96.0-97.0)	1.18 (1.16-1.21)	Reference Group
**GP Characteristics**					
Sex					p = 0.7678
* Missing*	*0*	*0*			
Male	583,200	6,984	60.5% (59.1-61.9)	1.20 (1.17-1.23)	Reference group
Female	388,900	4,562	39.5% (38.1-40.9)	1.17 (1.13-1.21)	0.99 (0.95-1.04)
Age					p = 0.4575
* Missing*	*6,400*	*83*			
<45 years	250,500	2,951	25.7% (24.5-27.0)	1.18 (1.13-1.23)	Reference group
45-59 years	473,400	5,544	48.4% (46.9-49.8)	1.17 (1.14-1.21)	0.99 (0.95-1.04)
60+ years	241,800	2,968	25.9% (24.6-27.2)	1.23 (1.18-1.28)	1.03 (0.96-1.10)
Practice location					p = 0.0024
* Missing*	*1,400*	*18*			
Major Cities	687,500	8,253	71.8% (70.5-73.1)	1.20 (1.17-1.23)	1.13 (1.04-1.22)
Inner Regional	187,800	2,134	18.3% (17.3-19.4)	1.14 (1.08-1.19)	1.04 (0.95-1.13)
Outer Regional/Remote	95,400	1,141	9.9% (9.0-10.7)	1.20 (1.12-1.28)	Reference group
Practice size					p = 0.0019
* Missing*	*18,900*	*234*			
Solo GP	103,600	1,427	12.6% (11.6-13.6)	1.38 (1.29-1.47)	Reference group
2-4 GPs	287,100	3,427	30.3% (29.0-31.6)	1.19 (1.15-1.24)	0.88 (0.82-0.96)
5-9 GPs	365,500	4,109	36.3% (34.9-37.7)	1.12 (1.09-1.16)	0.85 (0.78-0.92)
10-14 GPs	136,500	1,601	14.2% (13.1-15.2)	1.17 (1.11-1.24)	0.88 (0.80-0.96)
15+ GPs	60,500	748	6.6% (5.9-7.3)	1.24 (1.14-1.33)	0.87 (0.78-0.97)
Country of graduation					p = 0.7983
* Missing*	*2,700*	*31*			
Overseas graduate	297,300	3,659	31.8% (30.4-33.1)	1.23 (1.19-1.28)	1.01 (0.96-1.06)
Australian graduate	672,100	7,856	68.2% (66.9-69.6)	1.17 (1.14-1.20)	Reference Group
**Time**					p = 0.4451
Year					1.00 (1.00-1.01)

*CI* confidence interval

## Discussion

### General statement

Among the reported oral conditions, dental and mucosal-related conditions were the two most commonly managed types. Medications, referrals to dentists and surgeons, and counselling, advice or education were common management actions for oral conditions. To our knowledge, this nationwide study is the first to provide detailed information about patient and GP characteristics, encounters and management of oral conditions by GPs in Australia.

### Encounters

At Australian GP practices, oral conditions were managed at a rate of 1.19 per 100 encounters. This result of this study may not be directly compared to past UK and Canadian studies’ results on management of oral conditions in GP and physician practice setting due to differences in study design, however the potential disparities in these rates may be attributed to differences in healthcare systems [30]. While the UK, Canada and Australia provide universal medical healthcare for individuals, there are differences in how dental services are funded [31–33]. Public dental services in the UK are funded by government and provided by the National Health Service[31], while dental services in Canada, New Zealand and Australia incur solely private contributions and are funded by the Government for eligible individuals (e.g. low-income earners and pensioners) [2, 32, 33]. The Australian government, for example, introduced healthcare subsidies in the form of Health Care Concession Card (HCC), providing free public oral health services to those eligible to address the out-of-pocket costs as a major barrier to accessing oral health care in a high proportion of people aged 15 years and over [2]. Cost is therefore likely a reason for people to consult GPs for their oral conditions.

### Patient demographics

Deterioration in general health as individuals age may lead to adverse effects on oral health and the ability to attend health services [34]. The present study found that individuals aged 70 years or more attended GPs for the management of oral conditions at significantly lower rates than those aged 54 years or younger. This finding aligns with results of National Study of Adult Oral Health 2017–2018 which reported a decreasing trend in adults of older age groups reporting delays or avoidance of dental care due to costs, possibly attributed to a combination of greater capacity to afford dental treatments in older adults and public funded pensioners’ health care benefits [35]. Older Australians also present more frequently to GPs for general health conditions[36], which may in turn dilute the relative management rate of oral conditions among all health conditions presented at GP practices.

Global studies consistently show that individuals with disadvantaged SES are more likely to have poor oral health and associated pain compared to those with advantaged SES [37–40]. The cumulation of disadvantaged SES, costs for dental services and poor oral health may explain the higher GP management of oral conditions among patients from areas of disadvantaged SES. To reduce public health SES-related inequity, the Australian Government provides free public oral health services to those eligible via the aforementioned HCC [35]. However, HCC holders reportedly have poorer oral health than non-HCC holders attributable to financial barriers preventing access to private services and long waiting lists preventing access to public services [35]. As GP and ED services are more readily accessible, vulnerable Australians may instead seek management through these services instead.

There has been a rise in population of migrants from non-English speaking countries, whom oral health related behaviours and health literacy may be different to those raised in Australia [41]. Lower oral health literacy and poorer oral health were reported in migrant populations in Australia, Canada, and the US as a result of linguistic and cultural differences [41, 42]. Poorer oral health, lack of oral health literacy, cultural and linguistic diversities are possible justification for the higher likelihood of seeking non-oral health professionals such as GPs for management of oral conditions.

Similarly, Australian studies consistently reveal poorer oral health among Indigenous people compared to non-Indigenous peers [43–45]. This is likely to be correlated to social determinants of health among these communities reflected by the inequalities in education, jobs, and experiences of discrimination [46, 47]. As Indigenous Australians have been historically identified to be at risk of poor oral health, the Australian Government has provided health care subsidies and oral health outreach services to address these challenges [43].

### GP demographics

This study identified significantly higher likelihood of oral condition-related encounters at GP practices located in major cities and solo practices. This observation is unexpected as the dental practitioner to GP ratio per population size has been historically lower in regional or remote areas compared to major cities [48, 49]. Furthermore, financial barriers may be more prevalent among residents of rural areas compared to major cities, attributed to a higher proportion of people from low socio-economic backgrounds in rural areas [50]. The correlation between GP demographics and likelihood of managing oral conditions remains obscure and may need clarification in future studies.

### Oral conditions

Among the oral conditions managed by GPs, dental and oral mucosal related problems were the most prevalently reported in this study. These findings align well with a study of medical practitioners in the province of Ontario, Canada. This study identified diseases of the teeth and supporting structure, diseases of hard tissues, and diseases of oral soft tissues excluding lesions specific for gingiva and tongue as the most common oral conditions seen by a medical practitioner [12].

### Management

For the management of oral conditions, GPs prescribed antibiotics and analgesics, referred to a dentists or dental surgeon, and provided counselling, advice, or education. In general, most dental conditions require procedural treatments outside of GP’s scope of practice that involves extensive diagnostic procedures [51, 52]. In contrast, the prescription of antibiotics in the dental practice has been defined as low-value care, because antibiotics are rarely helpful with relieving the source of infection, symptoms or present harm to the population by antibiotic resistance, and introduces economical and pharmaceutical wastage [53]. The correct management of oral mucosal conditions, on the other hand, require accurate diagnostic skills and techniques acquired by education not included in medical education curriculum [54–56].

Despite the best intent in addressing patients’ oral conditions, the lack of confidence in managing oral conditions is commonly reported among GPs in Australia [57]. As oral conditions are commonly encountered in GP practices, integrating oral health education in medical curricula and continuous professional development of medical practitioners in diagnosis and management of common oral diseases may improve GP’s confidence and accuracy in managing oral conditions while facilitating timely referral to dental practitioners.

### Implications

This study showed that a proportion of patients are consulting GPs for management of oral conditions in Australia. Although the reason for seeing a GP instead of a dental practitioner for managing oral conditions was not explored in this study, past studies have identified high cost and low accessibility as barriers for Australians in accessing oral health care [5]. These barriers may explain the higher likelihood of having oral conditions managed by GPs in vulnerable population groups such as HCC holders and people from a non-English speaking background, disadvantaged SES background or Indigenous background. As there is a lack of public funding in oral health care in Australia, approximately 4 in 10 Australians aged 15 years and over avoided or delayed visiting a dental practitioner due to cost [58]. Furthermore, it should also be noted that more than four out of five dental practitioners work in a private care setting [58]. Australians who cannot afford or access oral health care may end up seeing a GP for management.

GPs may be exposed to patients who may require urgent management of oral conditions. During these appointments, GPs commonly prescribed medications, provided advice and referred patients to other health professionals such as dental practitioners. As GPs may be involved in oral health care in primary care settings, improving oral health training of GP may in turn improve GPs engagement in the management of oral conditions. Currently, there has been limited culture of collaborative practice between GPs and dental practitioners, which is essential in effectively mitigate oral health disparities [20, 59]. Despite evidence suggesting integrated healthcare model incorporating dental practitioners into primary care may lower overall healthcare cost[60], dental practitioners are frequently not included as a part of the healthcare team in primary care settings [61]. Frameworks to integrate dental care into primary medical care and standardised referral pathways from GP to dental practitioner may be developed to improve patients’ rate of dental attendance, which potentially leads to reduction of oral diseases and improvement of self-rated health [62].

### Limitations

Although the BEACH dataset provides a large sample size with linkage of GP’s management approach to the oral conditions, this study did not establish the linkage between the management approach and the conditions. While also possible, this study did not report the difference in GP management rates between new and old or recurrent oral conditions. Although oral problem or diagnosis coded via ICPC-2 PLUS were classified, definitions of ICPC-2 PLUS codes may overlap. The management of specific oral conditions (new and old) will be examined in subsequent studies. The results of this study are limited to Australian GPs and may not be generalised to other countries. Furthermore, this study has assumed the accuracy of the diagnosis made by the GPs as it is not possible to validate the accuracy.

### Future research

This study provides a snapshot of current state of oral condition-related encounters in the Australian general practice setting. Findings of this study pave the way for opportunities to improve value and quality of oral health care, and alleviate inequity in accessing oral healthcare. Future studies may be conducted using the BEACH dataset in the investigation of specific oral conditions and management approaches by GPs. Future studies may also explore reasons for higher likelihood of oral conditions to be managed in solo GP practices and GPs located in major cities. Public health initiatives can be developed in educating GPs and other primary care providers (such as nurses and allied health professionals) to recognise, manage, and facilitate timely referral of oral conditions to reduce low-value care and improve overall public health outcomes. Furthermore, policymakers may recognise disadvantaged populations and redirect oral healthcare access to individuals who are susceptible to poor oral health.

## Conclusion

This study provides a preliminary overview of management of oral condition-related problems by GPs based on patient and GP characteristics, specific oral conditions managed, and management approaches used by GPs. Patients with certain vulnerable demographics were more likely to attend a GP for management of oral conditions. Common oral conditions and management approaches among these encounters were identified. The study suggests that there is a potentially significant role for primary care practitioners such as GPs in providing oral health care. In addition to promoting oral health related training among GPs, collaboration between GPs and dental practitioners through integrated healthcare model and referral pathways are important to alleviate the current oral health disparities. The findings of this study contribute to public health and health policy discussions around optimising primary care provision in oral health.

## Supplementary Information

**Additional file 1:** **Appendix A.** Classification and subclassification of ICPC-2 plus code assigned oral health problem/diagnosis.

## Data Availability

The raw BEACH data are not publicly available due to the sensitivity around health data. Researchers with ethically appropriate research questions can request access to the data via the University of Sydney. They can contact Dr Christopher Harrison at christopher.harrison@sydnety.edu.au for further information about access.

## Abbreviations

AIHW: Australian Institute of Health and Welfare
ASGS: Australian Statistical Geography Standard
ATC: Anatomic Therapeutic Chemical classification
BEACH: Bettering the Evaluation and Care of Health study
CI: Confidence intervals
ED: Emergency department
GP: General practitioner
HCC: Health Care Concession Card
ICPC-2: International Classification of Primary Care—Version 2
IRSAD: Index of Relative Socio-Economic Advantage and Disadvantage
SES: Socioeconomic status
UK: United Kingdom
