# Supplementary material for: Encounters and management of oral conditions at general medical practices in Australia
Source: BMC Health Serv Res. 2022 Aug 8;22:1013. doi: 10.1186/s12913-022-08299-2 (PMC9361532; doi:10.1186/s12913-022-08299-2)
Supplement: Supplementary file 1 — Additional file 1: Appendix A. Classification and subclassification of ICPC-2 plus code assigned oral health problem/diagnosis. [file 12913_2022_8299_MOESM1_ESM.docx]

Appendix A: Classification and subclassification of ICPC-2 plus code assigned oral health problem/diagnosis.

|  | **Oral Health Problem/Diagnosis** | **ICPC-2 plus code** |
| --- | --- | --- |
| **DENTAL** | | |
| Infection or Disease | Abscess;dental | D82013 |
|  | Infection;dental | D82020 |
|  | Infection;tooth | D82017 |
|  | Dental caries | D82002 |
|  | Disease;teeth | D82004 |
| Pain or Symptoms | Pain;dental | D19032 |
|  | Toothache | D19008 |
|  | Pain;tooth/teeth | D19003 |
|  | Loose;tooth/teeth | D19014 |
|  | Sympt/complaint;teeth | D19011 |
|  | Problem;wisdom teeth | D19033 |
|  | Problem;dental | D19031 |
| Idiopathic Conditions | Teething | D19006 |
|  | Abnormality;dental | D82010 |
|  | Dental malocclusion | D82011 |
|  | Dental impaction | D82007 |
| **MUCOSA** | | |
| Fungal Infection | Thrush;oral | D83015 |
|  | Candidiasis;oral | D83004 |
|  | Moniliasis;oral | D83002 |
| Oral Ulcerations | Ulcer;mouth | D83036 |
|  | Ulcer;aphthous | D83018 |
|  | Ulcer;stomatitis;acute | D83003 |
|  | Burn(s);to;mouth | D83019 |
|  | Ulcer;tongue | D80006 |
|  | Ulceration;oropharynx | D83020 |
| Tongue disorder/ problem/symptom | Glossitis | D83010 |
|  | Geographic tongue | D83016 |
|  | Carcinoma;tongue | D77015 |
|  | Neoplasm malig;tongue | D77016 |
|  | Abscess;tongue | D83027 |
|  | Abnormal;taste | D20004 |
|  | Abnormal;colour;tongue | D20006 |
|  | Coated tongue | D20007 |
|  | Bleeding;tongue | D20010 |
|  | Pain;tongue | D20016 |
|  | Swollen;tongue | D20022 |
|  | Black;tongue | D20024 |
|  | Tongue tied | D81004 |
|  | Sympt/complaint;tongue | D20036 |
| Mouth disorder/ problem/symptom | Bad/foul;breath | D20001 |
|  | Drooling | D20003 |
|  | Bleeding;mouth | D20009 |
|  | Dry;mouth | D20014 |
|  | Halitosis | D20015 |
|  | Pain;mouth | D20017 |
|  | Bad;taste (in mouth) | D20020 |
|  | Dribbling;saliva | D20029 |
|  | Sympt/complaint;mouth | D20034 |
|  | Excessive;salivation | D20037 |
|  | Ludwigs angina | D83029 |
|  | Infection;mouth | D83037 |
|  | Burning (in);mouth | D20011 |
|  | Dysphagia | D21004 |
|  | Foreign body (in);mouth | D79005 |
|  | Lesion;mouth | D20038 |
|  | Choking | D21002 |
|  | Difficulty;swallowing | D21003 |
|  | Feeling (of);choking | D21005 |
|  | Pain;swallowing | D21010 |
|  | Problem;swallowing | D21007 |
|  | Unable (to);swallow | D21001 |
| Lip disorder/ problem/ symptom | Cracked;lips | D20002 |
|  | Abnormal;colour;lip | D20005 |
|  | Bleeding;lips | D20008 |
|  | Dry;lip | D20013 |
|  | Pain;lip | D20018 |
|  | Swollen;lip | D20021 |
|  | Rash;lip | D20030 |
|  | Sympt/complaint;lip | D20031 |
|  | Harelip | D81002 |
|  | Angular cheilosis | D83034 |
|  | Cleft;palate/lip | D81005 |
| Mucosal Condition of  unknown causes | Leukoplakia;oral | D83022 |
| Malignancy | Neoplasm malig;mouth/gum | D77014 |
| Other diseases | Stomatitis;acute | D83035 |
|  | Cheilitis | D83014 |
|  | Uvulitis | D83005 |
|  | Disease;tongue/mouth/lip | D83038 |
|  | Cyst;oral | D83028 |
| **PERIODONTAL** | | |
| Gingivitis | Gingivitis | D82006 |
| Periodontitis | Periodontitis | D82021 |
| Abscess | Abscess;gums | D82014 |
|  | Pyorrhoea alveolaris | D82008 |
| Pericoronitis | Operculitis | D82012 |
| Necortising ulcerative gingivitis | Vincents angina | D83032 |
| Other unspecified gum diesease | Infection;gum | D82016 |
|  | Disease;gums | D82003 |
| Other symptoms | Pain;gums | D19002 |
|  | Swollen;gum | D19007 |
|  | Bleeding;gums | D19001 |
|  | Retraction;gums | D19030 |
|  | Sympt/complaint;gums | D19010 |
| **TEMPOROMA NDIBULAR JOINT** | | |
|  | Problem;temporomandibular jt | L07004 |
|  | Dysfunction;temporomandibular | L07007 |
|  | Pain;jaw | L07001 |
|  | Sympt/complaint;jaw | L07003 |
|  | Grinding;teeth | D29002 |
|  | Trismus | L07006 |
| **ORAL GLANDS** | | |
|  | Parotitis | D83030 |
|  | Infection;salivary gland | D83011 |
|  | Calculus;salivary | D83013 |
|  | Blocked;salivary duct | D83006 |
|  | Sialoadenitis | D83026 |
|  | Neoplasm malig;parotid | D77005 |
|  | Carcinoma;parotid | D77011 |
|  | Mucocoele;mouth | D83031 |
|  | Mucocoele;salivary gland | D83023 |
|  | Disease;parotid glan ex mumps | D83024 |
|  | Disease;salivary glan ex mumps | D83025 |
|  | Swelling;parotid | D29015 |
|  | Swelling;salivary gland | D29019 |
| **TRAUMA** | | |
| Trauma to teeth | Fracture;tooth/teeth | D80007 |
|  | Injury;teeth | D80005 |
| Trauma to Jaw | Fracture;jaw | L76027 |
|  | Dislocation;jaw | L80007 |
|  | Injury;jaw | L81061 |
|  | Fracture;mandible | L76025 |
| Trauma to Tongue | Injury;tongue | D80002 |
| **NEURALGIA** | | |
|  | Trigeminal Neuralgia | N92001 |
| **DENTAL RELATED** | | |
| Dental related medication | Medication;prophylactic;dental | D44017 |
| Denture problem | Problem;dentures | D19013 |
|  | Effect(s);dentures | D19029 |
| Dental procedure | Dental procedure | D59008 |
|  | Check up;dental | D31003 |
|  | Surgery/extraction;dental | D54007 |
|  | Extraction;tooth/teeth | D52011 |
|  | Extraction;wisdom tooth/teeth | D52014 |
|  | Dental proc;root canal therapy | D59014 |
|  | X-ray;dental | D41012 |
| Dentist Referral | Referral;dentist | D66002 |
| Oral Health Advice | Advice/education;oral health | D45004 |
